# Natural History of Man

**Published:** 1848-10-01

**Authors:** 


Art. VII.—*The Natural History of the Human Species; its Typical forms, Primæval Distribution, Filiations, and Migrations.* By Lieut.-Colonel Charles Hamilton Smith, K.H. Edinburgh: W. H. Lizars. London: Samuel Highley. pp. 464. 1848.

The Ethnological Society have, from the first, put forward as their motto, and the recommendation of their claim to support, this assertion of Cuvier: "In every region of the earth man has, almost universally, been regarded by us with indifference. It is difficult to account for such neglect, but it is no less a misfortune to be deplored."

Much has indeed been done towards the vindication of the race of man from the opprobrium of a libellous orang-outan derivation; since Linnæus classed men with bats as well as monkeys, because—and only because—each of them have pectoral mammæ; since the same great naturalist failed to discover enough of anatomical distinction between man and the black pingo of Africa to prevent his classing them together as being of one species, and naming the one "homo sapiens" and the other "homo noylodytes."

The "Natural History of Man," by the learned Dr. J. C. Prichard, the President of the above-named Society, is too well known to need more than reference to its title here. The book to which we would now call the attention of our readers bears a very similar name: it is called "The Natural History of the Human Species." It is not the production of a doctor of divinity, laws, or physic; it is written by Lieut.-Col. Charles H. Smith, K.H., K.N., F.R.S. & F.L.S. It is ornamented by a portrait of the author for a frontispiece; but we feel quite sure, that a gentleman who has exhibited so much learning and research as are evidenced in the work before us, must possess a more intellectual countenance than "W. H. Lizars" has been pleased to exhibit in his sketch.

We will now proceed to give such a cursory view of the book as our limits will permit; premising that it contains, besides plates of skulls &c. of the leading varieties of the human race, ancient and modern, no fewer than thirty-four coloured plates of heads of every human diversity of colour and contour; from black to white, and "all the shades between;" from Caucasian oval to Mongolian triangle; from the negro facial angle of 70° to the European angle of 80°; from African wool, to the finest head of hair that ever delighted the eyes of a perruquier, or simulated the quondam *cheveleure* of any modern antique Apollo of a drawing-room.

Colonel Smith's main position is directly opposed to that of Dr. Prichard: he contends for the various origin of the human species. From the progressive development observable throughout nature, he inclines to think that the negro, as the most inferior race, was the first created; then the Mongolic; and lastly, the Caucasian.

He also insists much on his opinion, that some race of the genus homo was a contemporary with several extinct species of animals—an opinion to which, if much weight could be attached to the facts and authorities which he cites in sufficient numbers, he would almost convert us, did we not know that the first comparative anatomists and geologists of the day are entirely opposed to such a conclusion; we ourselves heard Professor Owen say, at the conclusion of the able course of lectures on warm-blooded animals, which he recently delivered in the theatre of the College of Surgeons, that not one authentic instance had ever come to his knowledge of the bones of man having been discovered in a truly fossil state; and he expressly referred to those accounts of the discovery of human bones in caves along with those of the hyæna, elephant, extinct carnivora, &c., which Colonel Smith quotes as undoubted facts, but which he considered as not less undoubted fables.

We cannot but remark, that we find in the course of this work a number of facts put forward as unquestioned, to which we feel irresistibly impelled to attach "a little crooked thing which asks questions." For example, in support of his opinion, that the southern and eastern shores of Asia are undergoing a continual depression and submergence under the sea, he says:—

"Since these lines were first written (1845), *if the foreign news may be credited*, an event of this kind has again taken place in the maritime provinces and the Yellow Sea, the waters rising in the Gulf of Pecheler, to the destruction of several hundred thousand human lives, innumerable cattle, the loss of all the houses and provisions, and the total ruin of above sixteen millions of the population, who were driven to seek shelter and food in the upland provinces."

He goes on to say:—

"Even admitting probable exaggeration in the report, it is an event far surpassing the traditional deluges of Greece, or any other recorded in profane history. It is an occurrence *that may boldly be claimed* as a proof of continued depression of the southern and eastern shores of Asia and the oscillations produced on the sea by submarine disturbance, which thus, like a great tide wave, passes upon the land far above its usual limit."

We take breath, after quoting this astounding account, determining henceforth to read the newspapers with greater attention, and not, if possible, again to be in ignorance for three long years after the event, that a deluge had taken place, at hearing of which Noah himself might have turned pale.

We pass, however, from the more strictly geographical and ethnological division of this work, to that which possesses a more psychological interest. We have said that the author inclines to the opinion that the three typical forms of man are distinct species, and not merely varieties of one aboriginal pair. He says (this quotation, by the way, is a fine specimen of his style):—

"It does not appear that a thorough research has yet been made in the successive cerebral appearances of the fœtus, nor of the character the brain of infants exhibits immediately after parturition, in each of the three typical forms. M. de Serres, indeed, has led the way, and already, according to him, most important discoveries have resulted from his investigations; for should the condition of cerebral progress be more complete at birth in the Caucasian type, as his discoveries indicate, and be successively lower in the Mongolic and intermediate Malay and American, with the woolly haired least developed of all, it would follow, according to the appointed general law of progression in animated nature, that both, or at least the last mentioned, would be in the conditions which show a more ancient date of existence than the other, notwithstanding that both this and the Mongolic are so constituted that the spark of mental development can be received by them through contact with the higher Caucasian innervation; thus appearing in classified zoology to constitute three species, originating at different epochs, or simultaneously in separate regions, while, by the faculty of fusion with the last or Caucasian, imparted to them, progression up to intellectual equality would manifest essential unity, and render all alike responsible beings, according to the degree of their existing capabilities—for this must be the ultimate condition for which man is created. Fanciful though these speculations may appear, they seem to confer more harmony upon the conflicting phenomena surrounding the question, than any other hypothesis that rests upon physiology, combined with geological data and known historical facts."

M. de Serres, as quoted by our author, has published a pretty complete theory of cerebral development, on the ascending scale: he continues—

"The higher order of animals, according to the investigations of M. de Serres, passes successively through the inferior state of animals, as it were *in transitu*, adopting the characteristics that are permanently imprinted on those below them in the scale of organization. Thus the brain of man excels that of any other animal in complexity of organization and fulness of development. But this is only obtained by gradual steps. At the earliest period that it is cognizable to the senses, it appears a simple fold of nervous matter, with difficulty distinguishable into three parts, and having a little tail-like prolongation, which indicates the spinal marrow. In this state it perfectly resembles the brain of an adult fish; thus assuming, *in transitu*, the form that is permanent in fish. Shortly after, the structure becomes more complex, the parts more distinct, the spinal marrow better marked. It is now the brain of a reptile; the change continues by a singular motion. The *corpora quadrigemina*, which had hitherto appeared on the upper surface, now passes towards the lower; the former is therefore a permanent situation in fishes and reptiles, the latter in birds and mammalia. This is another step in the scale. The complication increases, cavities or ventricles are formed, which do not exist in either fishes, reptiles, or birds. Curiously organized parts, such as the *corpora striata*, are added. It is now the brain of mammalia. Its last and final change is wanting, that which shall render it the brain of man, in the structure of its full and human development. But although in this progressive augmentation of organized parts, the full complement of the human brain is thus attained, the Caucasian form of man has still other transitions to undergo before the complete *chef d'œuvre* of nature is perfected. Thus, the human brain successively assumes the form of the Negroes, the Malays, the Americans, and the Mongolians, before it attains the Caucasian; nay more, the face partakes of these alterations. One of the earliest points where ossification commences is the lower jaw. This bone is therefore sooner completed than any other of the head, and acquires a predominance which it never loses in the Negro. During the soft pliant state of the bones of the skull, the oblong form which they naturally assume approaches nearly the permanent shape of the American. At birth the flattened face and broad smooth forehead of the infant, the position of the eyes, rather towards the sides of the head, and the widened space between, represent the Mongolian form, which, in the Caucasian, is not obliterated but by degrees, as the child advances to maturity."

We will conclude by extracting our author's summary of the foregoing facts and opinions. He says—

"Among these, perhaps not one is more forcible than the fact that the lowest form of the three is the most ready to amalgamate with the highest. Again, that both the beardless and woolly haired acquire the Caucasian expression of beauty from a first intermixture, and very often both stature and form excelling their type; and in the second generation, the eyes of Mongoles become horizontal, the face oval, the crania of the Negro stock immediately expand in their hybrid offspring, and leave more durable impressions than where the order is reversed. Even from the moment either typical stock is in itself in a position to be intellectually excited by education, it is progressive in its development in succeeding generations. Here, then, at the point of most intense innervation, the spark of indefinite progress is alone excited and communicated in power, precisely according to the quantity received. For the rest, gestation, puberty, and duration of life are the same; and in topographical location, though each is possessed of a centre of vitality, yet all have races and tribes scattered in certain directions through each other, and to vast distances, at the very first dawn of historical investigation."

This may be the cause why all nations acknowledge a great deluge, although they do not foresee a second. It is, however, true that the obvious inference to be drawn from the foregoing remarks does not amount to a demonstration that mankind is of one species only, . . . . . but that the intention of an aboriginal unity of the species is at least so far indicated by the circumstance of man's typical stock having all a direct tendency to pass upwards towards the highest endowed, rather than to a lower condition, or to remain stationary.

